# Reach adaption to a visuomotor gain with terminal error feedback involves reinforcement learning

**DOI:** 10.1371/journal.pone.0269297

**Published:** 2022-06-01

**Authors:** Tsuyoshi Ikegami, J. Randall Flanagan, Daniel M. Wolpert

**Affiliations:** 1 Mortimer B. Zuckerman Mind Brain Behavior Institute, Columbia University, New York, NY, United States of America; 2 Department of Neuroscience, Columbia University, New York, NY, United States of America; 3 Center for Information and Neural Networks (CiNet), National Institute of Information and Communications Technology, Suita City, Osaka, Japan; 4 Graduate School of Frontier Biosciences, Osaka University, Osaka, Japan; 5 Department of Psychology, Queen’s University, Kingston, Ontario, Canada; 6 Centre for Neuroscience Studies, Queen’s University, Kingston, Ontario, Canada; Curtin University, AUSTRALIA

## Abstract

Motor adaptation can be achieved through error-based learning, driven by sensory prediction errors, or reinforcement learning, driven by reward prediction errors. Recent work on visuomotor adaptation has shown that reinforcement learning leads to more persistent adaptation when visual feedback is removed, compared to error-based learning in which continuous visual feedback of the movement is provided. However, there is evidence that error-based learning with terminal visual feedback of the movement (provided at the end of movement) may be driven by both sensory and reward prediction errors. Here we examined the influence of feedback on learning using a visuomotor adaptation task in which participants moved a cursor to a single target while the gain between hand and cursor movement displacement was gradually altered. Different groups received either continuous error feedback (EC), terminal error feedback (ET), or binary reinforcement feedback (success/fail) at the end of the movement (R). Following adaptation we tested generalization to targets located in different directions and found that generalization in the ET group was intermediate between the EC and R groups. We then examined the persistence of adaptation in the EC and ET groups when the cursor was extinguished and only binary reward feedback was provided. Whereas performance was maintained in the ET group, it quickly deteriorated in the EC group. These results suggest that terminal error feedback leads to a more robust form of learning than continuous error feedback. In addition our findings are consistent with the view that error-based learning with terminal feedback involves both error-based and reinforcement learning.

## Introduction

Sensorimotor adaptation can be achieved through error-based learning or reinforcement learning, which are generally considered to involve different computations and have distinct neural underpinnings [1–4]. Whereas error-based learning is thought to rely on sensory prediction errors (e.g., a difference between the observed and predicted position of the hand), reinforcement learning is driven by reward prediction errors. In the field of motor control, these two forms of learning have been examined using reaching tasks in which participants are required to move a cursor to a target under a visuomotor transformation that alters the mapping between hand movement and cursor movement. In studies of error-based adaptation, visual feedback of the cursor may be continuous, provided throughout the movement, or terminal, only provided at the end of the movement. In studies of adaptation using reinforcement, the cursor is not visible and binary feedback (success/failure) about whether the cursor reached the target is provided.

Recent research has compared the stability of visuomotor adaptation when achieved through either continuous error-based feedback or binary reinforcement feedback [5, 6]. For example, Therrien and colleagues [6] adapted participants to a visuomotor rotation using either continuous visual feedback of the cursor or binary feedback at the end of the movement indicating whether or not the unseen cursor reached the target. Following adaptation, participants performed a hundred trials without any feedback. Whereas adaptation was maintained in participants who had received binary feedback, it quickly decayed in participants who had received continuous error feedback. This result suggests that binary reinforcement learning, unlike error-based learning with continuous visual feedback, results in more persistent changes in motor commands.

There is evidence indicating that error-based adaptation achieved through terminal feedback, in which the position of the cursor is only shown at the end of the reaching movement, may involve elements of reinforcement learning. Izawa and Shadmehr [7] compared adaptation, when reaching to a single target under a visuomotor rotation, in different groups who received either continuous error feedback, terminal error feedback, or binary feedback. Following adaptation, they examine perceived hand position when reaching to a training target and generalization of learning to targets in different directions. They found that generalization was more global under continuous error feedback compared to binary feedback with intermediate generalization under terminal error feedback. They also found that the remapping of hand position following adaptation under terminal error feedback was intermediate between the remapping observed under continuous error feedback and binary feedback, in which no remapping was observed. Based on these results, the authors concluded that adaptation with terminal error feedback is driven by both sensory prediction errors and reinforcement prediction errors.

Given the greater persistence of adaptation following binary reinforcement feedback compared to continuous error feedback [6], the hypothesis that adaptation with terminal error feedback partially involves reinforcement learning [7] predicts that adaptation with terminal error feedback should be more persistent than adaptation with continuous feedback. The main aim of this paper was to evaluate this prediction. Three groups of participants made reaching movements to a training target while a change in visuomotor gain was gradually implemented. The different groups received either continuous error feedback (EC), terminal error feedback (ET), or binary reinforcement feedback (success/fail) at the end of the movement (R). Following adaptation, we first examined generalization of adaptation to targets in different directions. Our aim was to test whether, using a different adaptation task, we would observe the same pattern of generalization reported by Izawa and Shadmehr [7]. Following the generalization phase, we tested the persistence of adaptation in the EC and ET groups when the cursor was extinguished and only binary reward feedback was provided.

We found that adaptation was more persistent in the ET group compared to the EC group. We also found that generalization was stronger in the R group compared to EC groups, with intermediate generalization observed in the ET group. These results provided support for the hypothesis that ‘error-based’ learning with terminal feedback involves reinforcement learning in addition to error-based learning.

## Materials and methods

### Participants

Thirty right-handed participants—18 females and 12 males aged 24.2 ± 4.5 years (mean ± s.d.)—with normal or corrected-to-normal vision took part in the experiment. The experiment was approved by the Institutional Review Board of the Columbia University Medical Center and conducted according to the Declaration of Helsinki. All participants gave written informed consent prior to participating and were naïve to the purpose of the study. Participants were randomly assigned to one of three groups (see below). One participant, in the EC group, was excluded from analysis because he frequently failed to move within the specified reach duration, and took three times as long as all other participants to complete the experiment. The sample size we selected was based on previous work in this field [e.g. 8–10] showing that 8–12 participants per group results in robust group effects, meaningful effect sizes, and effects that are observed in the majority of individual participants.

### Apparatus

**Fig 1:**
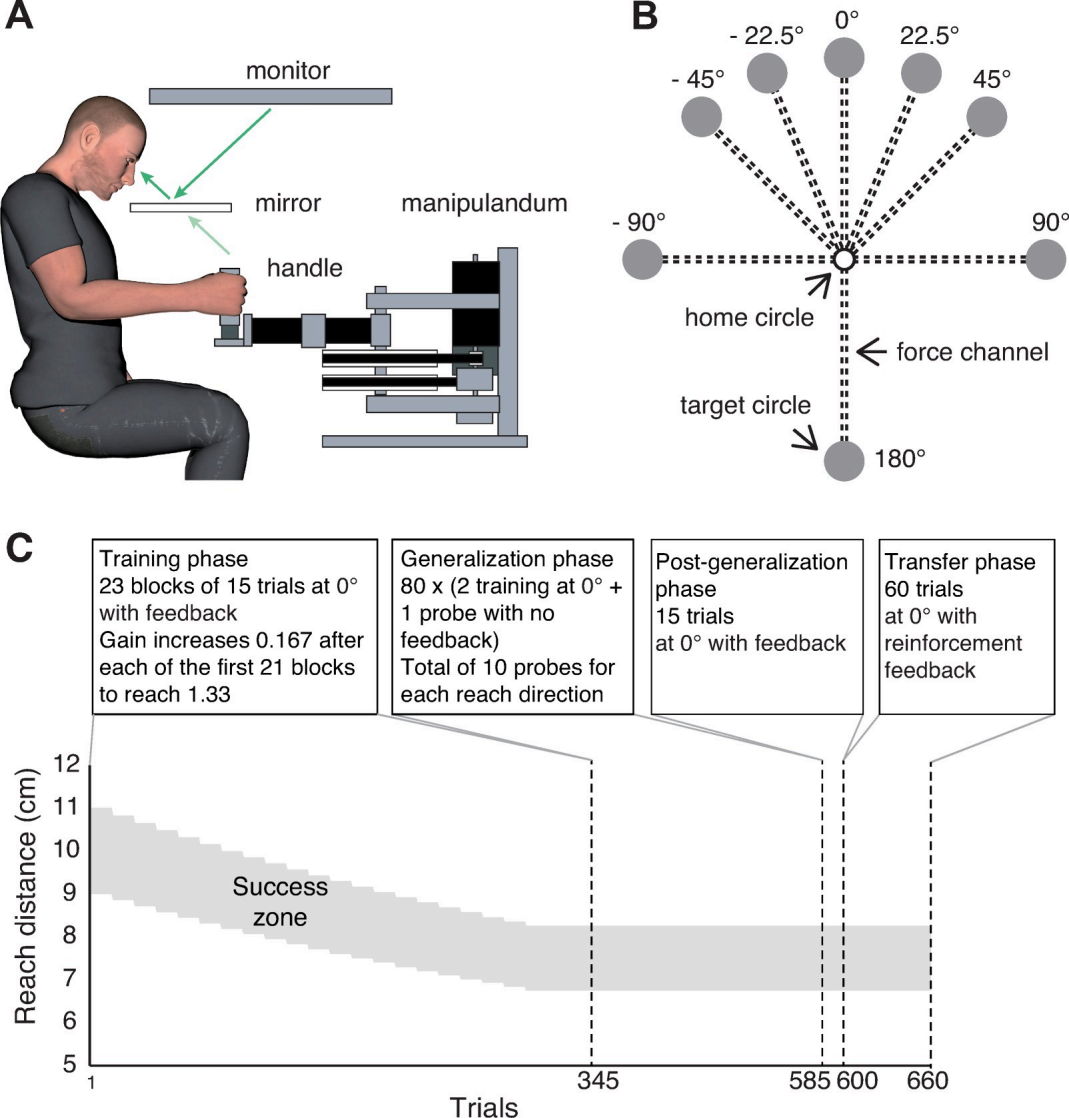
Experimental setup and paradigm. **(A)** Participants grasped the handle of a robotic manipulandum (vBOT) that could generate forces in the horizontal plane. Visual stimuli and feedback of movements were presented using a top-mounted computer monitor seen via a mirror. **(B)** Participants made a reaching movement for one of eight possible targets in each trial. The 0° target was used for both training trials (for adaptation to a novel gain) and probe trials (for evaluation of generalization function) while the other seven targets were used only for probe trials. All trials were performed under a force channel environment generated by vBOT. **(C)** Trial structure in the four phases of the experiment. The success zone shows the range of reach distances required for binary success feedback across the experiment.

Seated participants performed right-handed reaching movement while grasping the handle of a vBOT planar robotic manipulandum [11] that measured the position of the handle at a sampling rate of 1 kHz and could apply forces to the hand via the handle (Fig 1A). The right forearm was supported on an air sled, which constrained the hand and arm movement to the horizontal plane. Targets (1 cm radius disks), a home circle (0.5 cm radius disk), and a cursor (0.3 cm radius disk) controlled by the handle were displayed on a computer monitor, which was mounted horizontally above the vBOT. These visual stimuli were projected into the plane of hand movement via a mirror located halfway between the monitor and the handle. The mirror prevented participants from directly viewing their hand or the handle.

### Procedure

Participants made reaching movements to move the cursor from the home circle, positioned in the midsagittal plane ~35 cm in front of the chest, to one of eight possible targets (Fig 1B). The targets were located 10 cm away from the home circle and arranged at 0° (forward reaching direction in the midsagittal plane), ±22.5°, ±45°, ±90°, and 180° (positive angles defined as clockwise).

A visuomotor gain could be imposed between the radial displacement, from the home circle, of the cursor relative to the hand. For example, with a gain of 1.33, the participant would be required to shorten their hand reach distance to 7.5 cm (~10/1.33) to move the cursor to a target, located 10 cm away. With a gain of 1, the cursor is aligned with the hand position. Participants performed all trials under a force channel environment [12] where the hand was constrained to a straight-line path to the target by a spring-like force (spring constant of 3000 N m^-1^ and a damping coefficient of 5 Ns m^-1^) acting perpendicular to the straight line to the target. This ensured that any behavioral changes during gain adaptation were attributed to changes in the participant’s ability to control movement distance, rather than movement direction.

Each trial started when the cursor had remained within the home circle at a speed below 3 cm s^-1^ for 0.1 s. A target was then presented together with a tone indicating that participants should initiate a movement. If participants moved before the tone or took longer than 1.0 s to respond to the tone, they were required to repeat the trial. Participants were instructed to make a fast reaching movement so that the cursor ended in the target. The end of the movement was taken when the hand speed fell below 3 cm s^-1^ for 0.1 s. If participants took longer than 0.8 s to make the movement, they were provided with a “move faster” message and required to repeat the trial. After each movement, the vBOT passively moved the participant’s hand back to the home circle. During the passive movement, no visual feedback of the cursor was provided.

In the experiment there were two trial types. On training trials, the target was always at 0° and participants received feedback about their performance that depended on the group to which they were assigned. For the EC group, the cursor was visible throughout the movement. For the ET group, the cursor disappeared when the hand left the home circle and reappeared at the end of the movement at its final position for 0.2 s. For the R group, the cursor disappeared when the hand left the home circle and did not reappear. In all groups, if the center of the (seen or unseen) cursor landed within the target, the target ‘exploded’ into small fragments that scattering radially (lasting for 0.2 s) and a pleasant chime sound was played. Otherwise, the target remained intact with no sound presented to signify failure. Note that participants in the R group only received this binary feedback of success or failure.

In addition to these training trials, participants performed probe trials that were used to measure generalization of learning to the eight targets. In these probe trials, no feedback was provided. That is, the cursor was visible only at the home circle and no success or failure feedback was provided. These trials were indicated by a magenta target while for the other trial types a yellow target was presented.

Whereas previous studies examining how different forms of feedback influences learning have focused on adaptation to visuomotor rotations, we opted to examine adaptation to a visuomotor gain change for three reasons. First, we wanted to ensure that participants could successfully adapt their movement when only binary success or failure feedback was provided (i.e., the R group). A previous study reported that with such binary feedback, only two-thirds of participants successfully adapted to a gradually introduced 25° visuomotor rotation [13]. In our pilot experiments, we also observed that a similar fraction of participants failed to adapt to a gradually introduced visuomotor rotation (even when using a rotation angle of 15°). Second, a complication of examining generalization following adaptation to a visuomotor rotation is that, during adaptation, participants make reaching movements between the target direction and the required hand direction and therefore will have already made reaching movements to some generalization targets and not others. In contrast, as a gain change perturbs along the reach there is no such asymmetry. Finally, we think it is valuable to use a different type of perturbation so as to examine the generality of previous findings.

#### Trial structure

Participants first completed the training phase in which they completed 21 blocks of 15 training trials in which the gain was increased slowly from 1 to 1.33 in increments of 0.167 per block, followed by two additional blocks with the final gain. The training phase was followed by a generalization phase in which trials were performed as triplets with two training trials followed by a probe trial to one of the 8 targets. Ten blocks of 24 trials were performed with each of the 8 targets probed once in a pseudorandom order in each block. The generalization phase was followed by the post-generalization phase which consisted of 15 training trials.

The post-generalization phase was followed by a transfer phase in which the EC and ET group performed a block of 60 training trials with reinforcement feedback with the gain maintained at 1.33. A short rest break was given after the first 15 of these trials. For the R group, we tested transfer to continuous error feedback. As expected, this transfer was excellent and we did not report these data as they were not germane to our main hypothesis.

Prior to the experiment, all participants performed a familiarization session. This involved a total of 356 trials with the gain set to 1 and distributed across the eight reach directions. These trials included probe trials and training with all three types of feedback to ensure that all groups had the same experience before starting the experiment and were able to calibrate themselves to the baseline gain. To encourage participants to make similar baseline movements across all types of trials, the peak speed of the movement was required to fall in a range from 40 to 60 cm s^-1^. If the peak speed was outside the range, the participant was presented with a “too slow” or “too fast” message and was required to repeat the trial. Note that this speed constraint was imposed only in the familiarization phase. Participants then completed a practice session that was a shortened version of the main experiment (consisting of all trial types they would experience in the main session) and with a gain of 1 throughout. For each of the two feedback conditions that the participant experienced in the main experiment, there were 30 training trials followed by a generalization phase with 5 blocks of 24 trials.

### Data analysis

For each trial we calculated the gain of the movement as the ratio of the target distance (10 cm) to hand distance from the home circle at the end of the movement.

During the generalization phase each probe trial was preceded by two training trials which were included to maintain adaptation during this phase. However, participants sometimes failed to land on the target with the seen or unseen cursor on these training trials. Because we were interested in describing generalization of fully adapted behavior, we implemented the following procedure to remove trials where behavior was not fully adapted. For each triplet of the generalization phase (2 training trials followed by a probe trial), if the cursor on the second training trial failed to land on the target, the probe trial was excluded from the analysis. This criterion led to the exclusion of 6.3, 20.4, and 45.0% of the 80 probe trials from the EC, ET, and R groups, respectively. Although the R group had the greatest exclusion (range for this group 25–65% across participants), we had a sufficient number of trials for analysis. Specifically, in the generalization phase we included, on average, 5.5 trials out of the 10 probe trials in the R group. The number of included trials at the ‘most excluded target’ ranged from 1 to 7 across participants with a mean of 3.7. Note that the pattern of significance of the ANOVA, used to assess generalization, was the same when these data were not removed.

To analyze generalization, for each target we calculated the mean gain of the probe trials across repetitions for that target. The gain was then normalized to the gain of the probe trials at the training (0°) target so that values represent a proportion of the adaptation for the training target.

We examined differences in generalization across groups in two ways. First, we carried out a two-factor (group by target) mixed-design ANOVA. While the factor of groups had three levels: EC, ET, R, the factor of target included seven levels: ±22.5°, ±45°, ±90°, and 180°.

Mexican hat (Ricker wavelet) to the normalized gain change as a function of angular deviation from the training target:

$$g(\theta )=\alpha \left(1-{\left(\frac{\theta }{\sigma }\right)}^{2}\right){exp}^{-\frac{\theta^{2}}{2\sigma^{2}}}+(1-\alpha )$$

where *α* and σ are the amplitude and width (s.d.) of the generalization function, and *θ* is angular deviation from the training target. This function was fit to the data averaged across participants in each group by minimizing the mean squared error (MATLAB function, nlinfit). Four models were considered which each shared or did not share values of *α* and/or *σ* across groups. We initially fit a Gaussian generalization function to the data but, based on a reviewer’s comment, we used a Mexican hat function because it provided a better fit to the generalization data. Note that our key conclusions do not depend on whether we fit the generalization data with a Gaussian or Mexican hat.

## Results

**Fig 2:**
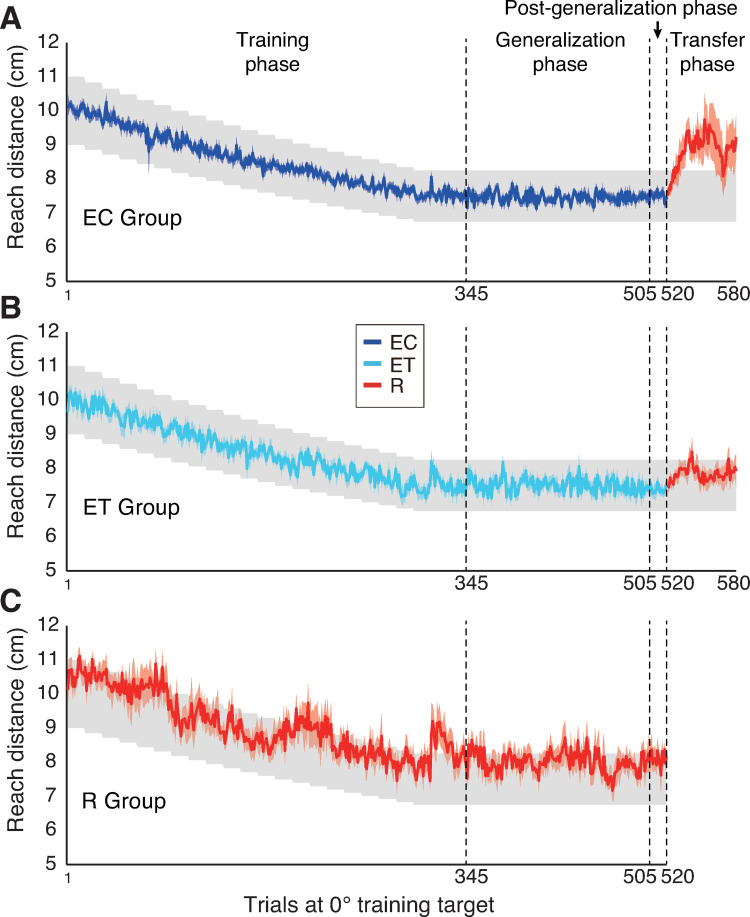
Time course of adaptation. (A-C) Hand reach distance in trials with the 0° training target, across the different phases of the experiment, for the EC, ET, and R groups. The colored lines and shading show the mean and SE for each group. The grey shading shows the reward zone.

Three groups of participants adapted to a gradually implemented change in visuomotor gain—from a cursor to hand movement gain of 1 to a gain of 1.33—while reaching in a horizontal plane to a straight ahead (0°) training target. Each group experienced one of three forms of feedback: continuous visual feedback of the cursor controlled by the hand (EC), visual feedback of the terminal position of the cursor at the end of the movement (ET), or target hit success or failure with no cursor feedback (R). Participants in all groups successfully adapted to this gain change by decreasing their hand reach distance across trials (training phase in Fig 2A–2C). To quantify adaptation we computed, for each participant, the average reach distance in all trials with the training target in which the gain was 1.33 (end of training phase and generalization phase), and compared this value to the reach distance in the first block of training trials (15 trials). Paired t-tests with Bonferroni corrections showed that for all three groups, reach distance decreased significantly (EC: t(8) = 62.15, p = 4.50e-12, *d* = 26.29; ET: t(9) = 30.61, p = 2.08e-10, *d* = 10.82; R: t(9) = 11.93, p = 8.09e-7, *d* = 3.97).

**Fig 3:**
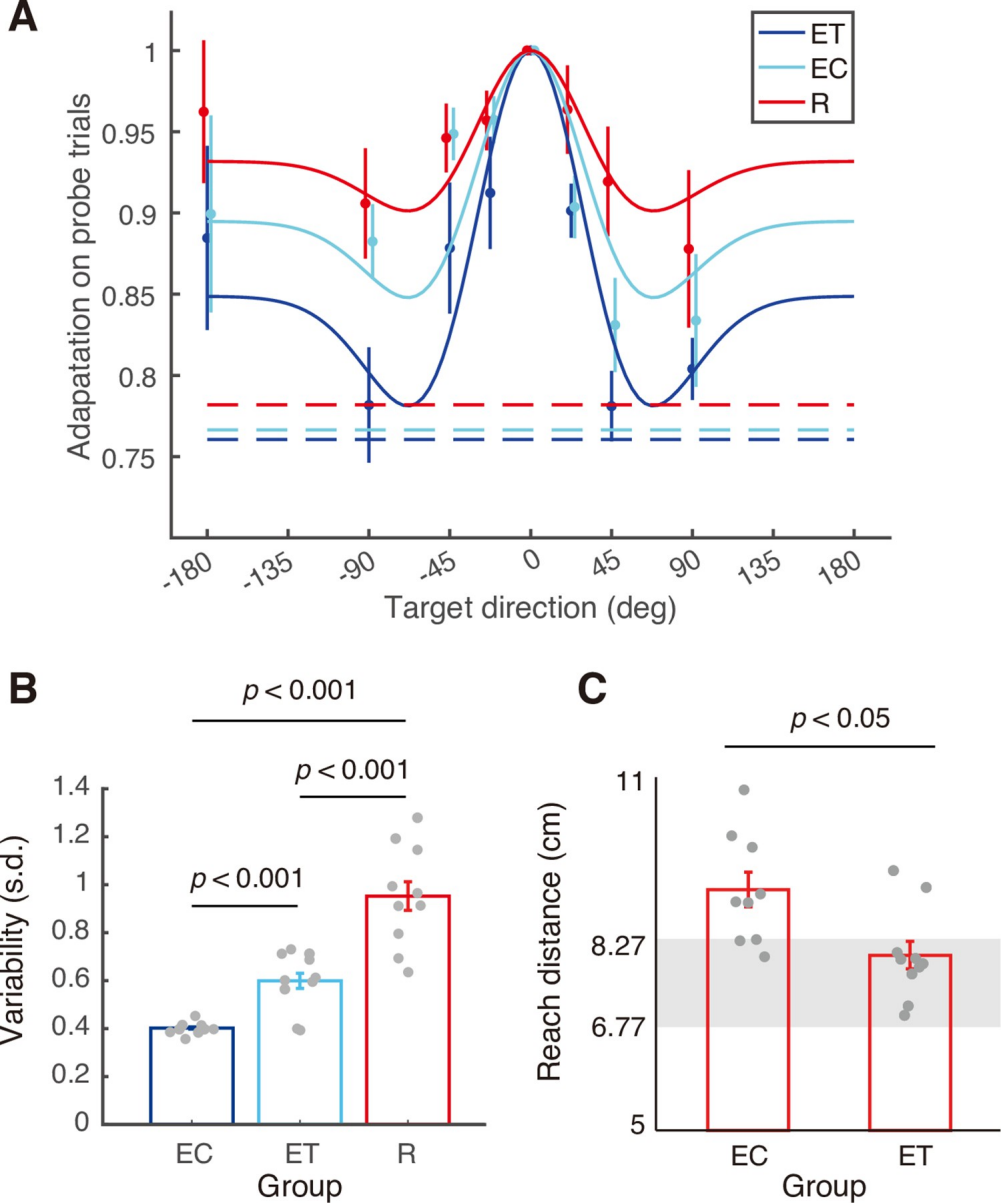
Generalization, variability and retention of adaptation. (A) Generalization on probe (no feedback) trials as a function of target direction. The adaptation for each participant was first averaged across repetitions for the same probe target, and then normalized to the adaptation for probe trials to the training target. Circles and error bars represent means ± SEs across participants. The solid lines show fits of the best fitting Mexican hat generalization functions (fixed width but varying amplitudes). The dashed lines show the level of adaptation corresponding to no generalization of the gain change. (B) Variability computed as the standard deviation of the reach distance of all training trials in the generalization and post-generalization phases where the cursor to hand movement gain was 1.33. Bars show mean ± SEs across participants. Dots show individual participants. (C) Hand reach distance in the second block of 15 trials in the transfer phase with reinforcement feedback for the EC and ET groups. Grey zones show the hand target region, which was constant (2 cm target diameter) in cursor coordinates. Bars and dots as in (B).

Following this adaptation, we tested generalization of learning by intermixing no feedback trials to targets in all 8 directions with training trials (with feedback) to the 0° target. The training trials were included to maintain adaptation. (Note that Fig 2A–2C only shows data for the training trials). Fig 3A shows the pattern of generalization for the three groups and the best fit Mexican hat model (see below). A two-way ANOVA showed main effects of group (F_2,26_ = 4.126, p = 0.028, η^2^ = 0.241) and direction (F_6,156_ = 6.78, p<0.001, η^2^ = 0.207) with no interaction (F_12,156_ = 0.679, p = 0.77, η^2^ = 0.050).

To test how generalization varied across groups, we fit a Mexican hat decaying generalization function (two parameters: *α*, the amplitude of decay from peak to asymptote and *σ*, standard deviation of the Mexican hat generalization) to the three groups. We compared four different models that varied in which parameters were shared across groups: a 2-parameter model with a single standard deviation, *σ*, and a single amplitude,

*α* (baseline model); a 4-parameter model with different *σ*’s for each group and a single single *α* (*σ* model); a 4-parameter model with a single *σ* and different *α*’s for each group (*α* model); and a 6 parameter full model with different *σ*’s for each group and different *α*’s for each group (full model). We found that the *α* model (F_2,20_ = 11.7, p = 4.3e-4, η^2^ = 0.539) but not the *σ* model (F_2,20_ = 0.90, p = 0.42, η^2^ = 0.083) provided a significantly better fit than the baseline model. Note that the likelihood ratio comparing the *α* model to the *σ* model was 16.8 in favor of the *α* model. We also found that the full model was not significantly better than the *σ* model (F_2,18_ = 0.17, p = 0.85, η^2^ = 0.019). These results indicate that the amplitude of decay of generalization varied across the three groups (*α* of 0.15, 0.11 and 0.07, for the EC, ET and R groups respectively) but that the width of generalization was similar (*σ* = 39.5°). Note that the generalization observed in the ET group was intermediate between the generalization seen in the EC and R groups.

To assess the variability in performance following adaptation, for each participant we computed the standard deviation of the reach distance of all training trials in the generalization and post-generalization phases where the cursor to hand movement gain was 1.33. The variability was 0.40 ± 0.03 cm (SE), 0.60 ± 0.12 cm, and 0.95 ± 0.21 cm in the EC, ET and R groups, respectively (Fig 3B). A one-way ANOVA revealed that the variability significantly varied across groups (F_2,26_ = 36.24, p = 3e-8, η^2^ = 0.736). Pairwise comparisons with Bonferroni corrections revealed that the variability in the R group was significantly larger than in the EC (t(17) = 7.76, p = 1.6e-6, *d* = 3.567) and ET (t(18) = 4.61, p = 6.5e-6, *d* = 2.062) groups, and the variability in the ET group was significantly larger than in the EC group (t(17) = 4.80, p = 5.1e-6, *d* = 2.203). Note that the variability observed in the ET group was intermediate between the variability seen in the EC and R groups.

After completing the generalization and the post-generalization phases, participants in the two error-based learning groups (i.e., EC and ET) then completed a transfer phase in which the gain was maintained at 1.33 and error feedback was replaced with reinforcement feedback. Importantly, participants in the reinforcement learning group (R) were able to adapt to the gain change and maintain this adaptation throughout the generalization phase. Thus, the hypothesis that learning with terminal error feedback shares components with reinforcement learning predicts that adaptation during the transfer phase should be more persistence in the ET group compared to the EC group. As shown in Fig 2A and 2B, participants in the EC group failed to maintain gain adaptation when reinforcement feedback was provided, whereas participants in the ET group did maintain the adaptation. More specifically, adaptation quickly decayed in the EC group and, on average, participants were not able to recover this adaptation when they received binary feedback indicating that the reach was unsuccessful. In contrast, adaptation decayed more slowly in the ET group and, on average, participants were able to recover when they received negative binary feedback. To quantify these effects, for each participant we computed the mean change in hand reach distance from the 15 trials of the post-generalization phase to the second block of 15 trials in the transfer phase (after which any drift in adaptation was largely completed). This change was larger (t_17_ = 2.524; p = 0.022, *d* = 1.224) for the EC group than and the ET group. We also directly compared the two groups on the second block of 15 trials in the transfer phase (Fig 3C). The hand reach distance was greater (t_17_ = 2.833; p = 0.011, *d* = 1.374) in the EC group (in which 6 of 9 participants exhibited means outside the reward zone), than the ET group (in which 2 of 10 participants exhibited means outside this zone).

## Discussion

The aim of this paper was to test the hypothesis that sensorimotor adaptation with terminal error feedback involves elements of reinforcement learning, driven by reward prediction errors, in addition to error-based learning, driven by sensory prediction errors. Using a visuomotor gain task, we compared error-based learning with continuous feedback (EC), error-based learning with terminal feedback (ET), and reinforcement learning with binary feedback (R). We found that when error-based feedback was removed following adaptation, learning persisted in the ET group—as in the R group—but not in the EC group. We also found that both the spatial generalization of learning and the variability of adapted performance in the ET group was intermediate between the EC and R groups. All three of these findings support the idea that error-based learning with terminal feedback is driven, in part, by reward prediction errors (i.e., reinforcement learning).

Although the majority of studies examining motor learning have focused on error-based learning, a growing number of motor learning studies have investigated reinforcement learning [5–7, 14–22]. Although error-based and reinforcement learning are generally considered to involve different computations and engage different neural mechanisms [1–4], Izawa and Shadmehr [7] provided evidence that error-based learning with terminal feedback has commonalities with reinforcement learning. Using a visuomotor rotation task, these authors found that, in comparison to error-based learning with continuous feedback, both error-based learning with terminal feedback and reinforcement learning resulted in weaker spatial generation, increased variability of adapted performance, and far less (if any) change in the perceived position of the hand.

Our study provides further support for the hypothesis that error-based learning with terminal feedback involves reinforcement learning. As observed by Izawa and Shadmehr [7], we found that both trial-by-trial variability and spatial generalization of adapted performance in our ET group was intermediate between our EC and R groups. However, whereas this previous study found that generalization was weaker in reinforcement learning than error-based learning with continuous feedback, we found the opposite effect in our task. The better generalization we observed with reinforcement learning may reflect the use of an explicit strategy (e.g., aim short of the target) that participants were able to partially generalize across target directions. Error-based learning with terminal feedback may also encourage the use of such an explicit strategy [17, 23, 24]. Consistent with this notion, a number of studies have shown that after-effects are weaker when adaptation is driven by terminal error feedback in comparison to continuous error feedback [23–26]. Our results are also broadly consistent with work showing that when both continuous error feedback and reinforcement feedback are provided during reach adaptation, continuous error feedback dominates [21]. That is, in the EC condition, learning may be largely driven by sensory prediction errors—with either a small or negligible contribution from reward prediction errors—whereas, in the ET condition, the contribution of reward prediction errors may be larger such that both sensory and reward prediction errors drive learning.

Note that in all three of our experimental groups, we observed partial generalization of gain adaptation in all directions. This finding contrasts with a previous study on gain adaptation in reaching which suggested full and uniform generalization across reach directions [27]. We do not have an explanation for this difference but note that our study and the previous study differed in two important ways. First, in our study visual feedback was provided in the horizontal plane of movement, whereas in the previous study visual feedback was provided on a vertical screen and not in the horizontal plane of movement. There is evidence that an incongruency between the plane of hand movement and the plane of visual feedback adds complexity to the movement planning process [28]. Second, the gain perturbation in the previous study was introduced instantaneously whereas in our study it was introduced gradually. Numerous studies have shown that the rate at which a perturbation is introduced can strongly influence motor memory formation [29–32]. According to a recent model, a new motor memory is formed in response to a rapidly introduced perturbation whereas an existing motor memory is adapted for a gradually introduced perturbation [33]. Not only did we observe a decrease in generalization away from the training direction, we also observed an ‘uptick’ in generalization in the polar opposite direction to the training direction. Such an uptick, which was well fit by a Mexican hat function, has been observed in previous studies of sensorimotor adaptation [e.g., 34, 35]. This suggests that that learning may be linked to the *axis* of movement and not just the direction.

Movement planning and control is thought to involve the specification of control policies that determine how sensory feedback about errors is transformed into corrective motor commands [36–38]. In error-based learning with continuous visual feedback, errors can be corrected ‘online’ during the movement (as well as between movements) and involve implicit remapping between commands and predicted sensory outcomes [39]. In contrast, in error-based learning with terminal feedback, as in reinforcement learning with binary feedback, corrections are generated between movements and appear to involve explicit learning of new action-outcome relationships [5–7]. Note that the two error-feedback groups (ET and EC) also received binary reward feedback on the training trials of the training and generalization phases. Thus, this reward feedback alone cannot account for the reinforcement learning we observed in the ET group.

In summary, our results support the hypothesis that error-based learning with terminal feedback involves reinforcement learning. We show that this finding, established using a visuomotor rotation task [7], generalizes to a different motor task involving gain adaptation. In addition, we provide support for the hypothesis using a different measure of adaptation; i.e., the persistence of adaptation.
