# Correction: Reach adaption to a visuomotor gain with terminal error feedback involves reinforcement learning

**DOI:** 10.1371/journal.pone.0308510

**Published:** 2024-08-02

**Authors:** Tsuyoshi Ikegami, J. Randall Flanagan, Daniel M. Wolpert

There is an error in the legend of Fig 3A located in the upper right of the image. The “ET” should have been light blue and “EC” should be dark blue. Please see the correct Fig 3 here.

**Fig 3:**
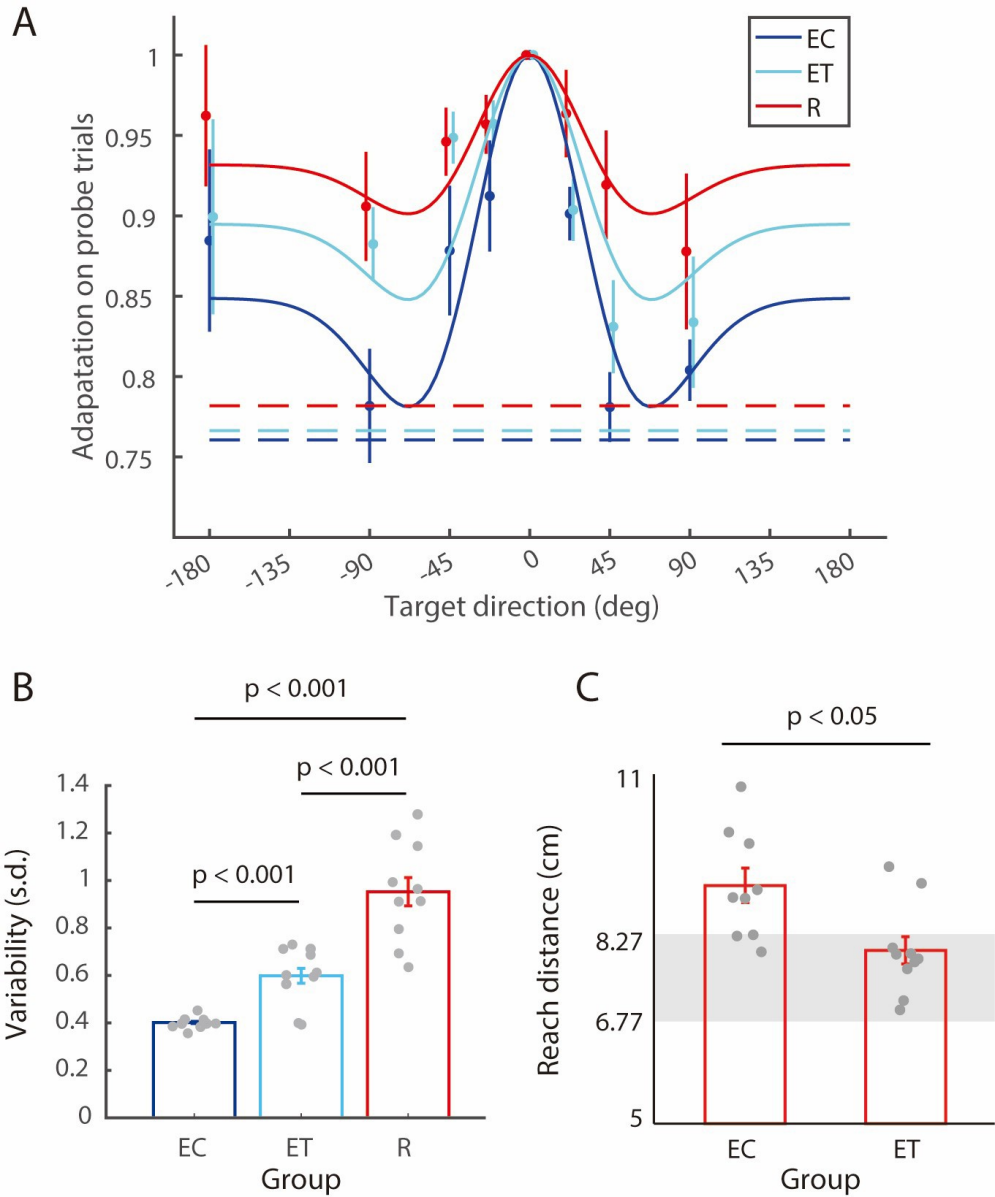
Generalization, variability and retention of adaptation. (A) Generalization on probe (no feedback) trials as a function of target direction. The adaptation for each participant was first averaged across repetitions for the same probe target, and then normalized to the adaptation for probe trials to the training target. Circles and error bars represent means ± SEs across participants. The solid lines show fits of the best fitting Mexican hat generalization functions (fixed width but varying amplitudes). The dashed lines show the level of adaptation corresponding to no generalization of the gain change. (B) Variability computed as the standard deviation of the reach distance of all training trials in the generalization and post-generalization phases where the cursor to hand movement gain was 1.33. Bars show mean ± SEs across participants. Dots show individual participants. (C) Hand reach distance in the second block of 15 trials in the transfer phase with reinforcement feedback for the EC and ET groups. Grey zones show the hand target region, which was constant (2 cm target diameter) in cursor coordinates. Bars and dots as in (B).
